# Ambulation in Dogs With Absent Pain Perception After Acute Thoracolumbar Spinal Cord Injury

**DOI:** 10.3389/fvets.2020.00560

**Published:** 2020-08-26

**Authors:** Melissa J. Lewis, Nick D. Jeffery, Natasha J. Olby, The Canine Spinal Cord Injury Consortium (CANSORT-SCI), Sarah A. Moore

**Affiliations:** Author Affiliations: DACVIM-Neurology, Associate Professor, Neurology and Neurosurgery, Department of Veterinary Clinical Sciences, The Ohio State University College of Veterinary Medicine, Columbus, OH, United States; DACVIM-Neurology, Professor of Neurology/Neurosurgery; Distinguished Chair of Gerontology, Department of Clinical Sciences, North Carolina State University College of Veterinary Medicine, Raleigh, NC, United States; DACVIM-Neurology Professor; Chair, and Head, Department of Small Animal Clinical Sciences, College of Veterinary Medicine and Biomedical Sciences, Texas A&M University, College Station, TX, United States; DAVCIM (Neurology), Assistant Professor of Neurology, Purdue University College of Veterinary Medicine, Department of Veterinary Clinical Sciences, West Lafayette, IN, United States; Professor Neurology & Neurosurgery; Professor in Small Animal Clinical Sciences, College of Veterinary Medicine, Texas A&M University, College Station, TX, United States; ACVIM - Neurology Professor and Service Head, Neurology and Neurosurgery, Department of Veterinary Clinical Sciences, College of Veterinary Medicine, The Ohio State University, Columbus, OH, United States; Department of Clinical Sciences, Colorado State University, Fort Collins, CO, United States; Department of Clinical Science and Services, Royal Veterinary College, Hatfield, United Kingdom; The Royal Veterinary College, University of London, Hatfield, United Kingdom & CVS Referrals, Bristol Veterinary Specialists at Highcroft, Bristol, United Kingdom; Institute of Veterinary Pathology, Faculty of Veterinary Medicine, Leipzig University, Leipzig, Germany; Division of Clinical Neurology, Department for Clinical Veterinary Medicine, Vetsuisse Faculty, University of Bern, Bern, Switzerland; Department Small Animal Medicine and Surgery, University of Veterinary Medicine Hannover, Hannover, Germany/Europe; Neurology and Neurosurgery, Department of Veterinary Medicine and Surgery, University of Missouri, 900 E. Campus Dr., MU Veterinary Health Center, University of Missouri, Columbia, MO, United States; Department of Small Animal Medicine and Surgery, University of Veterinary Medicine Hannover, Hannover, Germany/Europe; ^1^Department of Veterinary Clinical Sciences, Purdue University College of Veterinary Medicine, West Lafayette, IN, United States; ^2^Department of Small Animal Clinical Sciences, Texas a & M College of Veterinary Medicine and Biomedical Sciences, College Station, TX, United States; ^3^Department of Clinical Sciences, North Carolina State University, Raleigh, NC, United States

**Keywords:** spinal walking, deep pain negative, intervertebral disc herniation, canine, locomotion, gait generation

## Abstract

Acute thoracolumbar spinal cord injury (SCI) is common in dogs frequently secondary to intervertebral disc herniation. Following severe injury, some dogs never regain sensory function to the pelvic limbs or tail and are designated chronically “deep pain negative.” Despite this, a subset of these dogs develop spontaneous motor recovery over time including some that recover sufficient function in their pelvic limbs to walk independently without assistance or weight support. This type of ambulation is commonly known as “spinal walking” and can take up to a year or more to develop. This review provides a comparative overview of locomotion and explores the physiology of locomotor recovery after severe SCI in dogs. We discuss the mechanisms by which post-injury plasticity and coordination between circuitry contained within the spinal cord, peripheral sensory feedback, and residual or recovered supraspinal connections might combine to underpin spinal walking. The clinical characteristics of spinal walking are outlined including what is known about the role of patient or injury features such as lesion location, timeframe post-injury, body size, and spasticity. The relationship between the emergence of spinal walking and electrodiagnostic and magnetic resonance imaging findings are also discussed. Finally, we review possible ways to predict or facilitate recovery of walking in chronically deep pain negative dogs. Improved understanding of the mechanisms of gait generation and plasticity of the surviving tissue after injury might pave the way for further treatment options and enhanced outcomes in severely injured dogs.

## Terminology

- “Deep pain negative”: term synonymous with “absent pain perception” and defined as an absent behavioral response to noxious stimulation caudal to the injury level. For thoracolumbar SCI, this refers to absent pain perception to a mechanical stimulus in the medial and lateral toes of both pelvic limbs and base of the tail; in dogs, this term is applied in the acute setting with concurrent paraplegia to imply a functionally complete injury though sensory and motor status should be considered separately in the chronic setting.
- Sensorimotor complete injury: term used to describe functionally and/or physically complete injury in people where there is absent voluntary movement or pain perception below the injury level; synonymous with AIS-A designation using human SCI grading parameters.
- Ambulatory: the ability to rise and take at least 10 consecutive weight bearing steps unassisted without falling.
- Chronically paralyzed: broad, non-specific term used to capture the population of dogs with permanent neurologic impairment (motor, sensory, and/or deficits in continence) following severe SCI. Dogs in this group can exhibit paraplegia (i.e., no pelvic limb movement at all) or display varying degrees of pelvic limb movements that fall short of being useful (i.e., they remain non-ambulatory).
- Spinal walking: independent ambulation in a “deep pain negative” dog typically characterized by lack of coordination between thoracic and pelvic limbs, difficulty turning, or going backward, intermittent falling (especially when changing directions), frequently intact toe knuckling response but absent hopping, and increased spasticity.

## Introduction

The majority of dogs suffering from acute spinal cord injury (SCI) will recover adequate or even normal function (1). However, a subset of dogs with severe injury fail to regain pain perception caudal to the injury level (“deep pain negative”), remain incontinent and are classified as having an unsuccessful outcome (1–3). The permanent lack of pain perception has been commonly, and frequently incorrectly, interpreted as an indication of spinal cord transection, complete disconnection from all supraspinal influence and minimal to absent chance of meaningful recovery of function. However, a proportion of permanently deep pain negative dogs demonstrate notable spontaneous motor recovery over time (2, 4, 5). This can range from non-purposeful kicking movements of the limbs, especially following tactile stimulation below the injury level, to overground walking with minimal apparent paresis or ataxia. Ambulation exhibited by this population, typically known as “spinal walking,” is commonly considered exclusively reflexive stepping generated by the spinal cord caudal to the level of injury as described by experimental studies of SCI in dogs and other species (6–14). While relatively autonomous circuits within the spinal cord are integral, gait generation is a complex process with extensive coordination between various components of the central and peripheral nervous systems. Understanding the ways in which this circuitry is altered and also how it can recover after injury has broad therapeutic and translational implications.

This review will provide a comparative overview of locomotion and explore the physiological underpinnings of “spinal walking” after severe SCI in dogs. Additionally, the clinical characteristics of motor recovery with absent pain perception as well as proposed means to predict and facilitate its development in this population will be described.

## Comparative Review of Locomotion/Gait Generation

Normal locomotion is a complex action that involves coordination of multiple brain regions, circuitry within the spinal cord and peripheral nerves and muscles. The basic components of locomotion are evolutionarily conserved with broad overlap even between invertebrate and vertebrate animals. While motor systems within the brain and spinal cord are essential to producing locomotion, integration of sensory input at all levels is also integral to proper functioning and modulation of locomotion in response to environmental surroundings.

Within the brain there are several motor regions from which upper motor neurons arise to produce the descending motor tracts, with some variability in their relative importance across species. These include the primary motor cortex located in the parietal lobe of each cerebral hemisphere, the red nucleus of the midbrain and the reticular formation of the pons and medulla oblongata. Additionally, the mesencephalic locomotor region located just ventral to the caudal colliculi is involved in initiating stepping movements. Axons of neurons from this area do not directly project to the spinal cord but rather interact with other brainstem motor regions, especially within the reticular formation, to produce locomotion. All of these components are also influenced and modulated by the cerebellum and basal nuclei. Input from these areas allow for complex movements and adjustment of locomotor activity. The overall output of the brain activates spinal cord motor circuitry and produces voluntary motor activity (15–18).

Axons of the upper motor neurons (UMN) in the various motor regions of the brain form the descending motor tracts to the spinal cord. These descending motor tracts produce both inhibitory and excitatory influence on spinal cord interneurons and lower motor neurons (LMN) to initiate and regulate voluntary movement. These include the lateral and ventral (the latter being more developed in primates) corticospinal tracts, rubrospinal tract, and pontine and medullary reticulospinal tracts. While the vestibulospinal tracts play a crucial role in posture and influence locomotion, they will not be discussed in detail. The corticospinal tract originates in the primary motor cortex, follows the major descending white matter pathway (internal capsule, crus cerebri, pyramids) to the medulla where the majority of fibers cross at the pyramidal decussation to descend in the lateral funiculus of the contralateral spinal cord. It is primarily involved with complex and precise movements although is reported to retain a role in overall gait generation (17, 19, 20). The rubrospinal tract originates in the red nucleus, immediately crossing midline to travel in the contralateral lateral funiculus of the spinal cord. The pontine and medullary reticulospinal tracts start in the ill-defined reticular formation of the brainstem before descending in the ipsilateral ventral and lateral funiculi, respectively. The rubrospinal and medullary reticulospinal tracts facilitate flexor muscles and inhibit extensors while the pontine reticulospinal tract does the opposite, providing a tonic balance between facilitation and inhibition of spinal cord lower motor neurons (15–18). Direct evidence in dogs is sparse, but it has been demonstrated in cats that the reticulospinal pathways play an important role in postural control and basic gait generation on a flat surface while the rubrospinal tract is involved in both normal control of locomotion and in producing adaptive movements to changes in the environment (19, 21). The corticospinal tract is less well-developed in domestic species (compared to people and non-human primates) and is not considered essential to generate basic locomotor rhythms; however, it functions in parallel with the other motor pathways to primarily regulate and fine tune movements (19–21).

Within the spinal cord, circuitry involved in gait generation has been identified in multiple species and is known as the central pattern generator (CPG) (13, 16, 18, 22–24). The CPG organizes the basic pattern for stepping, independent of supraspinal or sensory input. This basic rhythmic pattern of the CPG is produced by interconnected, alternating, and mutually inhibitory flexor and extensor interneurons (25). This network is thought to extend the length of the spinal cord but has been most extensively studied in the lumbar region in relation to control of the pelvic limbs (in quadrupeds, or legs in people). In this context, it is located in the intermediate zone of spinal cord gray matter although the precise cranial to caudal location of integral components of the circuitry within the lumbar spinal cord might vary between species (e.g., cranial lumbar in dogs, rats, people; mid-caudal lumbar in cats) (24, 26). These interneurons, in turn, activate lower motor neurons *via* additional intermediary interneurons, the output from which serves as the final common pathway to produce locomotion *via* direct innervation of appendicular muscles (25). The CPG also provides coordination between left and right limbs via integration of commissural interneurons and thoracic and pelvic limbs, important in normal quadrupedal locomotion (15, 18, 27, 28). While autonomously capable of relatively complex patterns of activity, under normal (non-injured) conditions, supraspinal input is necessary for activation (29). Additionally, modifying input to the CPG is necessary to allow adaptation of the basic alternating stepping pattern. Sensory input derived from visual information, vestibular input, and both exteroceptor and proprioceptors located on the body and limbs is also an important component of locomotion, specifically providing information needed to adapt locomotion to an animal's surroundings (16).

Gait generation itself consists of two major phases, the postural stance phase and the protraction or swing phase. However, based on the activation pattern of specific pelvic limb muscles, the step cycle should really be considered as having four phases: flexion and first extension occur during swing while second and third extension occur during stance (30, 31). Second extension happens during the early part of the stance phase when the knee and tarsus joints actually flex despite contracting extensor muscles as the animal prepares to bear weight (30, 31). Third extension is characterized by hip, knee and tarsus extension as the weight of the body is pushed forward (30, 31).

## Plasticity of Locomotor Systems After SCI

The central nervous system is largely considered to have poor regenerative capacity; however, remarkable plasticity is possible. In fact, much of what is known about the organization and function of locomotor systems has been elucidated *via* various experimental spinal cord transection and decerebrate animal models (6, 7, 9–14, 32). Reorganization and adaptations that occur at all levels might influence recovery of motor function below the level of severe injury. These include regrowth of axons across the epicenter, recovery/reactivation of conduction of residually intact UMN axons traversing the lesion epicenter, a more autonomous role for the CPG, alterations in excitability of interneurons and LMNs below injury, activation of silent synapses, changes in synaptic weight, and alterations in sensory input or how afferent input is integrated at the level of the spinal cord below injury (29, 33–38).

Axonal regeneration of UMN axons has been demonstrated via experimental transection models although the capacity for regeneration varies between axon types and is limited compared to axons in the peripheral nervous system (37, 39–41). While serotonergic axons have demonstrated robust sprouting ability after injury, there are substantial deterrents to meaningful regrowth of most other disrupted axon systems (41). These include the size of the defect, astroglial scar formation, growth inhibitory molecules (e.g., chondroitin sulfate proteoglycans) and myelin-based growth inhibition (37, 39, 40). Additionally, there is no guarantee that regenerating axons will reconnect with the appropriate below-injury targets. These factors lead to minimal functional recovery in most complete transection models. There is active research regarding how to facilitate more effective regrowth through the use of various grafts, scaffolds, inhibitors of scar formation and other modulators of axonal growth (37, 39, 40, 42–47).

Fortunately, even with severe injury, physical spinal cord transection is uncommon. Residually intact, small diameter, subpial UMN axons traversing the lesion epicenter have been shown in various animals and people with functionally complete injury (33, 35, 48, 49). While the degree of loss of large diameter axons and abnormal myelination of residual fibers contribute to persistent neurologic deficits in chronic SCI, there is evidence of reactivation of surviving long tract axons within rubrospinal and other descending motor tracts (50, 51). This might serve to reestablish supraspinal influence on spinal cord circuitry and LMNs and contribute to recovery of voluntary motor control (35, 50). Prior work in rats and cats has shown that as little as 5–10% of the original population of axons can allow voluntary ambulation after severe injury (33, 50, 51).

Additionally, collateral sprouting of spared UMNs and regrowth of local propriospinal fibers traversing the site of injury have each been shown in experimental injury in rodents and lampreys (52–55). These mechanisms serve to produce novel, multisynaptic pathways, and reestablish the connections between UMNs and LMNs with associated improvements in motor function (39, 52). Interestingly, propriospinal neurons have also been shown to activate CPGs, highlighting their potential importance in achieving useful locomotor recovery after severe injury (39, 55–57).

Below the level of injury, notable changes also occur. There is increased importance of the integration between sensory input and CPG activity to coordinate motor output due to limited or lack of supraspinal control (29). Alterations in both motor neuron pool excitability and sensory input to the dorsal horn occur and likely contribute to functional status after injury (38, 58–62). For example, pharmacologic inhibition of post-synaptic inhibition with strychnine has been used to facilitate spinal walking in experimentally transected dogs (63). However, maladaptive plasticity and development of aberrant neuronal circuits commonly manifested as neuropathic pain or spasticity can also occur and impair functional recovery (58, 60).

## Spinal Walking Definition and Brief Overview

Dogs with chronic, permanent (i.e., more than 3 months after injury) loss of pain perception following acute severe, naturally-occurring thoracolumbar SCI are generally considered to have a limited capacity for locomotor recovery. Despite this presumption, a proportion of these dogs regain the ability to walk independently (2, 4). Unassisted ambulation in dogs chronically lacking pain perception (“deep pain negative”) has commonly been referred to as “spinal walking.”

Dogs without pain perception that exhibit such walking tend to show a spastic pelvic limb gait in which the stepping pattern of the pelvic limbs is not apparently coordinated with the thoracic limbs (the step cycles are out of phase) (Supplementary Materials 1–3). There is a tendency to fall to one side, especially when turning. Some dogs will exhibit “attempts” to correct the falling due to excessive spasticity in the limb ipsilateral to the fall. However, other dogs demonstrate the ability to walk much longer distances without falling. Limb movements during ambulation are variable; dragging of the toes is observed in some animals, but many also show excessively high stepping associated with dramatic flexor spasticity, especially when changing directions. It is also common for dogs to lean forward to facilitate standing up when initiating ambulation. This population commonly demonstrates intact toe knuckling response but very delayed to absent hopping, absent extensor postural thrust and inability to step backwards. Spinal reflexes are typically hyper-reflexive, flexor, and extensor spasticity are common. Chronic reflex perturbations are also common including an abnormal crossed extensor reflex between pelvic limbs (stimulating flexion in one pelvic limb that elicits reflex extension of opposite pelvic limb in a non-weight bearing position) and the presence of a “mass reflex” (simultaneous, below-injury movements including flexor spasms of the limbs, tail flagging, and evacuation of the bladder or colon elicited by tactile or other sensory stimulation such as manual bladder expression).

Spinal walking has been proposed to reflect reflexive stepping generated autonomously at the level of the spinal cord CPG in the absence of any supraspinal input (8, 16). This is supported by experimental transection models in dogs showing recovery of treadmill and over ground ambulation in the months after injury with similar electromyographic patterns to normal walking dogs (11, 13, 63–65). While Liu et al. found that no transected dogs without additional therapeutic intervention (polyethylene glycol at the site of transection) regained any pelvic limb motor function, they were only followed for two months which is likely premature to its typical development (65). Other work has showed spontaneous recovery of ambulation in a majority of dogs by an average of four months after transection without any specific therapy (11).

While preservation and/or effective reorganization of the CPG circuitry is integral to motor output after SCI, there are distinct differences between experimental and naturally-occurring SCI as well as between treadmill walking and over ground walking (2, 66). Importantly, simple activation of exclusively CPG-induced reflexive stepping post-injury might not adequately explain the broad variability in when and in which dogs develop independent, over ground ambulation despite persistently absent pain perception. In the normal, uninjured state, supraspinal input is considered necessary for initiation and control of voluntary over-ground locomotion mammals (16). Whether this requirement for supraspinal input to produce functional ambulation is maintained following severe SCI remains uncertain. In one study evaluating electrophysiologic evidence of long tract function in a group of dogs lacking pain perception, results suggested that recovery of supraspinal connections and walking were independent of each other (5). In another study of dogs with permanently absent pain perception, all dogs that recovered ambulation were noted to have a voluntary tail wag within one-month post-injury (2). This finding demonstrated intact brain to tail connections traversing the site of some so-called complete injuries and implied a potential association between such translesional connections and recovery of walking (2). While there is conflicting evidence regarding the role of supraspinal influence in severely injured dogs, there are also other factors to consider such as maintenance of certain sensory input that are likely crucial to guide the appropriate CPG-directed motor output in the post-injury setting (16, 29, 67).

Overall, development of ambulation in pain perception negative dogs likely reflects a reorganized CPG in complex coordination with multiple other factors that might include some degree of spared supraspinal influence, a certain threshold of motor neuron pool excitability, appropriate peripheral sensory input, activity specific locomotor training and yet to be determined combinatorial therapeutic interventions (4, 5, 68, 69).

## Clinical Description In Dogs With Naturally Occurring Injury

Ambulation in pain perception negative dogs secondary to naturally occurring injury is reported to range from 10 to 59%, with the large discrepancy likely due, in part, to differences in the patient population, the injury itself, and variable definitions of walking and pain perception (2, 4, 5, 69). Although it can be seen with a number of causes of acute SCI, the majority of what we know about this population comes from dogs that suffered intervertebral disc herniation (IVDH), the most common cause of acute SCI in dogs. While an association between injury type and development of spinal walking has not been identified, it appears to be less common in dogs who suffered vertebral column trauma (2, 4, 69). This might reflect that a large percentage of dogs are euthanized at the time of traumatic injuries due to poor prognosis relative to IVDH, but differences in the impact of injury type (e.g., higher rate of more extensive or multiple injuries and physical spinal cord transection in traumatic injuries) on locomotor systems is also possible.

The timeframe during which ambulation develops is also variable. In Olby et al. 2003, 7/18 (38%) dogs with absent pain perception secondary to IVDH regained ambulation on average over 9 months with a range of four to 18 months (2). Among a cohort of 94 dogs examined in the chronic setting in which nine were ambulatory with absent pain perception, the median time since injury at examination was 12 months (range of 3–89 months) (5). While time to develop ambulation was not specifically reported for the nine dogs, the overall timeframe is similar to Olby et al. In contrast, Gallucci et al. found median time to regain ambulation was just 75 days and ranged from 16 to 350 days for the 48/81 (59%) dogs with functionally complete injuries who walked again (4). Differences in study design likely contributed to this discrepancy. Most notably, dogs with shorter average time to ambulation underwent early post-injury, intensive rehabilitation which might have positively impacted recovery (4).

Development of ambulation in pain perception negative dogs is typically considered to require intact local reflex arcs to the pelvic limbs (i.e., an injury level cranial to the fourth lumbar vertebrae) to provide appropriate muscle tone and necessary weight bearing ability (8, 14). However, the importance of lesion location within the T3-L3 spinal cord region remains unclear. It has been suggested that lesions cranial to the thoracolumbar junction might impair supraspinal postural control of epaxial muscles and therefore prevent functional manifestation of the reflexive stepping, even if such spinal circuitry is intact (8). On the contrary, it has also been proposed that more cranial lesions (cranial to L2) might facilitate its development due to sparing of the intrinsic circuitry of the CPG integral to pelvic limb locomotor function (70, 71). The most common site among deep pain negative dogs who walked in one study was T12-T13 and ranged from T4-5 to L2-3 (4). No association between lesion location and ambulation has yet been identified (4, 5, 72).

Body weight but not body condition score has also been reported to influence development ambulation in pain perception negative dogs, with smaller dogs being more likely to become spinal walkers (4). The role of body weight distribution is unclear but compensatory forward loading on to the thoracic limbs has been demonstrated in dogs with SCI (73–75). It is possible that smaller dogs more effectively shift weight off of their pelvic limbs making it easier to “stand” and for stepping movements to become functional walking compared to larger, heavier dogs. The impact of limb length has not been specifically investigated. Anecdotally, taller dogs with a higher center of gravity are less likely to regain ambulation, perhaps due to greater demands on supraspinal postural control to maintain balance which might be lacking after severe injury. Deficiencies in lateral stability have been demonstrated in dogs with both complete and incomplete SCI and might support postural control as an additional factor contributing to return of functional ambulation beyond just regaining pelvic limb stepping movements (76). Clinically, lateral instability can be noted in this population as a tendency to ambulate reasonably well in straight lines but falling when attempting to turn or change directions. Younger age has also been suggested to promote its development (4). Other patient factors that logically might negatively influence motor recovery include lack of behavioral motivation, limb contractures, and severe limb muscle atrophy.

Among chronically deep pain negative dogs, clinical examination of spasticity has also been described in relationship to motor function (77). A canine spasticity scale was developed that specifically quantifies duration of patellar clonus and degree and duration of pelvic limb flexor spasms induced by pin prick to the bottom of the paw. The overall spasticity scale score and duration of flexor spasms were each positively associated with gait scores (77). While spasticity is typically considered a maladaptive response to severe injury in people resulting in pain, reduced quality of life and inconsistent impacts on daily functioning, its potential role in recovery of motor function is poorly understood (77–82). However, the data in dogs suggests that development of flexor spasms might indicate increased excitability of the intraspinal circuitry and improved recovery of stepping (77). Cutaneous sensory stimulation of the hind quarters after injury (especially of the perineum, tail, and paw) has also been suggested to produce stepping movements in dogs (11). The importance of afferent input has been demonstrated in cat and rodent models where sural nerve stimulation, tail electrical stimulation or manual tail or perineum manipulation enhanced pelvic limb stepping (6, 12, 26, 83, 84). In humans with incomplete SCI, cutaneous plantar sensory stimulation during motor training increased spinal cord excitability and has been suggested as a means to enhance recovery of motor function (85). Additionally, it has been advocated to incorporate a variety of walking surfaces for incomplete injury patients supporting an integral role for sensory input in promoting locomotor recovery (86). Although the role of targeted sensory input on the development of spinal walking has not been prospectively evaluated in dogs with naturally occurring injury, providing different sensory environments (e.g., grass versus hard surface flooring) and targeted afferent stimulation might be useful to facilitate walking in this population.

Electrodiagnostic testing has also been utilized to try to shed light on the long tract and local spinal pathways involved in the development of ambulation in dogs with absent pain perception (5, 68, 87). Evaluation of spinal cord long tracts utilizing transcranial magnetic stimulation (TMS) and cortical and spinal cord somatosensory evoked potentials (SSEPs) have produced conflicting results (5, 68, 87). In Lewis et al., no SSEPs traversing the injury site were identified but pelvic limb motor evoked potentials (MEPs) following TMS were noted in 4/20 dogs (including 3/5 ambulatory dogs) (68). Trans-lesional motor conduction was associated with higher open field gait scores and ambulation. One of the four dogs included in this group had present but blunted pain perception which supports a less severe injury and might have explained the MEP and recovery of ambulation (68). In contrast in Hu et al. 2018, cortical SSEPs and MEPs were noted in 12/34 (0/9 spinal walkers) and 19/85 (1/9 spinal walkers) chronically injured dogs, respectively, but no relationship was identified between the presence of either SSEPs or MEPs and ambulation (5). It is possible that trans-lesional conduction in chronic SCI provides insufficient influence in some injuries or is unrelated to the reorganization of spinal cord circuitry that produces walking. Clarification of these electrodiagnostic results and the role of residual or reestablished supraspinal input on long-term recovery of function below clinically complete injuries requires further study.

Local spinal reflex circuitry aimed at evaluating motor neuron pool excitability has also been evaluated in chronically injured dogs using the H-reflex (68). The H-reflex was present recording from the plantar interosseus muscles following tibial nerve stimulation in 19/19 of chronically injured dogs compared to 3/6 controls, and the H-reflex threshold (stimulus intensity at which the waveform first appeared) was lower in SCI dogs than in controls (68). This lowered threshold supports increased motor neuron pool excitability below injury compared to healthy animals without SCI. Notably, the H-reflex threshold was also inversely associated with open field gait scores among the dogs with chronic SCI (68). This suggests that increased motor neuron pool excitability might also play an important role in motor recovery following severe injury.

Magnetic resonance imaging (MRI) features of dogs with chronic SCI have also been described in relationship to below-injury functional status (72, 88, 89). On conventional MRI performed in the chronic setting, a longer length of apparent complete parenchymal compromise (i.e., no normal tissue discernible on consecutive transverse images at the lesion epicenter) was inversely associated with open field gait scores (72). Similarly, more extensive chronic intramedullary lesions or cavitations have been associated with failure to regain ambulation by 7 months after presentation (88). Diffusion tensor imaging (DTI), an MRI application in which images are derived from the cellular motion of water, and associated tractography, which provides a visual representation of spinal cord white matter tracts, have also been evaluated in this population (89). Decreased anisotropy at the lesion epicenter (i.e., loss of directional dependence of water diffusion which is high in the normal spinal cord) as measured by the DTI parameter, fractional anisotropy, and complete loss of fiber tracts traversing the site of injury on tractography were each inversely associated with gait scores (89). Interestingly, of the four deep pain negative dogs that were reported to have no trans-lesional fibers on tractography (two secondary to IVDH and two following vertebral column trauma), none was independently ambulatory. These findings suggest a role for supraspinal input in motor recovery after severe injury in at least some animals but the numbers were small and results require validation in a larger population of dogs.

## Prediction and Facilitation of Spinal Walking

While a variety of factors have been associated with the development of ambulation in dogs with absent pain perception, no predictors in the acute or subacute stage of its subsequent development have yet been established. Considerations worthy of further investigation include clinical parameters such as the onset of spasticity, imaging biomarkers such as DTI indices and tractography, electrodiagnostic evaluation of descending motor tract function or motor neuron pool excitability and serum and cerebrospinal fluid biomarkers of inflammation or structural spinal cord proteins (5, 68, 77, 89–98). While specific markers in serum and cerebrospinal fluid have not been evaluated to predict spinal walking, serum glial fibrillary acidic protein (GFAP) and phosphorylated neurofilament heavy chain (pNFH) have been reported to be useful among deep pain negative dogs in predicting outcome and the development of progressive myelomalacia (96–98). Biomarkers as potential prognostic indicators have been described in detail in the companion article in this issue, “Prognostic Factors in Acute Intervertebral Disc Disease,” and it is possible some of these will be useful in this population.

There is also currently limited evidence for specific treatments to facilitate the recovery of ambulation in dogs lacking pain perception. However, a variety of therapeutic interventions have been investigated in experimental models and human SCI to optimize recovery that might prove useful in this population. These include task-specific physical rehabilitation, functional electrical stimulation and epidural stimulation, targeted somatosensory stimulation, treating neuropathic pain, and other pharmacologic interventions (12, 26, 42, 70, 71, 99–108). Importantly, there is growing evidence that multimodal approaches to facilitate motor recovery might prove most useful in improving outcomes in conjunction with traditional approaches directed at the lesion epicenter (36, 70, 109, 110). This is supported by work in chronically sensorimotor complete people and rodent models in which epidural stimulation aimed at motor networks below the level of injury produced some voluntary control of limb function perhaps by unmasking limited residual supraspinal connections (71, 101, 106, 111). Thus, epidural stimulation with locomotor training efforts might be enhanced by combining them with strategies that also promote tissue level recovery at the site of injury. Additionally, combination therapy with task-specific training and chondroitinase ABC in experimental SCI models has been shown to promote regeneration and synergistic plasticity with a greater degree of effective synaptic connections reestablished below injury in an activity dependent manner (112–114). Chondroitinase therapy alone has been shown to be effective in dogs with chronic SCI including recovery of ambulation in 10%, the effect of which might be enhanced by combining it with other treatment modalities (115).

Among dogs lacking pain perception, early in-patient rehabilitation has been suggested as one factor that positively impacted the recovery of ambulation (4). Further evaluation of specific rehabilitation protocols, focusing on specific components of gait re-training, is warranted. Potassium channel antagonist, 4-aminopyridine, has also been demonstrated to improve ambulation in a subset of chronically paralyzed dogs (69, 116). While promising, the intrinsic value of such therapies as chondroitinase, rehabilitation, or 4-aminopyridine cannot be determined without widespread clinical use in this population. Epidural stimulation or functional electrical stimulation have not been evaluated in dogs with spontaneous SCI, but these techniques might prove useful and preliminary work to develop such devices are underway (117). Overall, exploring multimodal therapeutic approaches will likely prove most useful in enhancing motor recovery after severe, spontaneous SCI in dogs.

## Author Contributions

ML, NJ, and NO substantially contributed to manuscript concept, preparation, and editing. The additional members of the CANSORT-SCI^*^ contributed to manuscript concept, editing, and review. All authors contributed to the article and approved the submitted version.

## Conflict of Interest

The authors declare that the research was conducted in the absence of any commercial or financial relationships that could be construed as a potential conflict of interest.
